# Temporal trends in the utilization, costs, and outcomes of concomitant left atrial appendage closure across a statewide collaborative

**DOI:** 10.1016/j.xjon.2024.10.030

**Published:** 2024-11-14

**Authors:** Yas Sanaiha, Bob Kiaii, Jack C. Sun, Michael Madani, Tom C. Nguyen, Richard J. Shemin, Peyman Benharash, University of California Cardiac Surgery Consortium

**Affiliations:** a Division of Cardiac Surgery, University of California Los Angeles, Los Angeles, Calif; b Division of Cardiothoracic Surgery, Department of Surgery, University of California Davis Medical Center, Sacramento, Calif; c Division of Cardiothoracic Surgery, University of California Irvine, Orange, Calif; d San Diego Health System, Division of Cardiovascular and Thoracic Surgery, University of California, La Jolla, Calif; e Department of Surgery, Division of Cardiothoracic Surgery, University of California San Francisco, San Francisco, Calif

**Keywords:** left atrial appendage closure, postoperative atrial fibrillation, coronary artery bypass grafting, valvular heart disease, sex-based disparities, variation

## Abstract

**Objective:**

With the rising incidence of atrial fibrillation, left atrial appendage closure (LAAC) at the time of cardiac surgery remains an important adjunct. The present study characterized trends, associated resource utilization, and potential disparities in the use of left atrial appendage exclusion.

**Methods:**

Using a Society of Thoracic Surgeons regional academic collaborative database, we queried all adult patients undergoing coronary and valve procedures with concomitant LAAC between 2015 and 2021. Trends in LAAC, as well as the technique for closure, were evaluated. Multilevel hierarchical logistic modeling was applied to delineate factors associated with LAAC, accounting for patient and operative characteristics. Generalized linear models were developed to perform risk-adjusted incremental cost analysis.

**Results:**

Of the 8699 patients who met the study criteria, 1377 underwent left atrial appendage closure. Over the study period, the annual rate of LAAC increased from 16.7% to 30.8% (*P* < .001). LAAC patients were older, but less commonly insulin-dependent diabetics or on dialysis. Female sex, redo, and urgent operative status had lower risk-adjusted odds of LAAC. Although LAAC was associated with longer bypass time, there was no significant association with 30-day mortality or 30-day readmission. LAAC was associated with an incremental increase in adjusted costs by $10,602 (95% confidence interval, $4078-$17,126).

**Conclusions:**

Rates of LAAC are increasing but less common among female patients, as well as those requiring urgent/emergent interventions. LAAC did not significantly impact short-term mortality. Our results suggest that LAAC may be a high-value intervention among patient populations that have the greatest potential to derive its benefits.

## Graphical abstract

**Figure:**
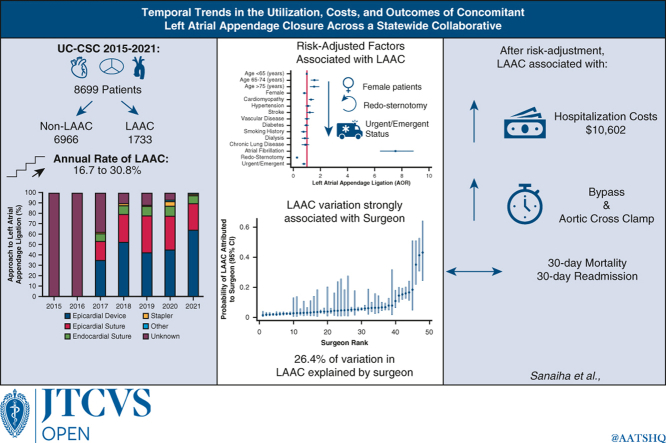

**Figure:**
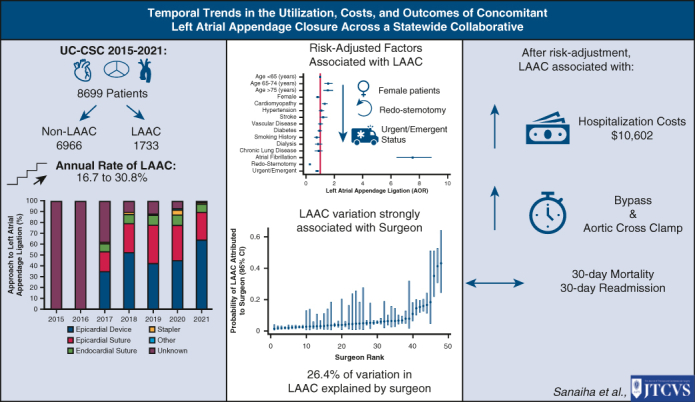
Rates of LAAC have increased, yet females are less likely to receive concomitant LAAC.

**Central Message**

Although associated with increased costs, there is no significant increase in risk-adjusted odds of mortality or major morbidity with concomitant left atrial appendage closure.

**Perspective**

Given the increasing prevalence of atrial fibrillation and growing evidence of reduction in associated thromboembolic complications with left atrial appendage closure (LAAC), consideration for expansion of this procedure is warranted. Dedicated evaluation of risk-adjusted lower odds of undergoing concomitant LAAC in female patients is warranted.

Atrial fibrillation (AF), the most common clinically significant cardiac arrhythmia, is associated with significant cardiovascular morbidity.^1^ Studies on the global burden of AF have shown that its prevalence to have doubled from 1990 to 2019.^1^ Importantly, development of AF is associated several acquired cardiac conditions including hypertensive, valvular and atherosclerotic heart diseases. Attributed to the increased inflammation and catecholamine levels in the postoperative period, de novo AF can develop in 30% to 50% of patients following cardiac operations. Thus, surgical management of AF and its sequelae has garnered much attention and remains a subject of debate.^2^

Recent societal guidelines have recommended surgical exclusion of the left atrial appendage (LAA) during cardiac operations in patients at high risk for thromboembolism (CHADS-VASC2 score >2).^3^ These guidelines have been supported by the Left Atrial Appendage Occlusion III (LAAOS III) trial, which demonstrated a reduced risk of stroke or systemic embolism with concomitant closure of the LAA during cardiac surgery, as well as by numerous meta-analyses.^4^, ^5^, ^6^ Despite purported benefits of LAA closure (LAAC), prior literature encompassing a myriad of obliteration techniques, has called into the question this benefit in patients without preexisting AF.^7^ One such meta-analysis of 3897 patients, stratified by preoperative AF status, found a greater stroke risk reduction in patients with preoperative AF.^7^ In light of the various LAAC methods available, the randomized ATLAS (AtriClip Left Atrial Appendage Exclusion Concomitant to Structure Heart Procedures) trial demonstrated a high rate of successful appendage exclusion using a commercial epicardial device, with a low incidence of related serious adverse events.^8^ The limited duration of follow-up in the ATLAS trial has been addressed by the ongoing Left Atrial Appendage Exclusion for Prophylactic Stroke (LeAPPS) trial, which is powered to evaluate event rates for thromboembolism.^8^^,^^9^

Given the ongoing controversy surrounding LAA management, we aimed to characterize modern trends of LAAC in a statewide academic cardiac surgery consortium. We hypothesized an increasing utilization of LAAC and the presence of institution- and surgeon-level variation in its use in patients with or without preexisting AF. We also examined associated factors, as well as financial and clinical outcomes of LAAC.

## Methods

Data for the present study were obtained from the University of California Cardiac Surgery Consortium (UCCSC) repository. Founded in 2013, the UCCSC is a collaborative encompassing 5 academic hospitals across California. Data elements, including those submitted to the Society of Thoracic Surgeons (STS), are collected prospectively and linked to financial data in compliance with policies of individual institutions and approved by the University of California System-Wide Review Board (IRB 16-000558; renewed April 17, 2024). Patient consent for the publication of study data was waived due to deidentified data collection.

The study cohort comprised all adult patients undergoing isolated coronary artery bypass grafting (CABG), isolated valve, CABG/valve, or multivalve operations between 2015 and 2021. Patients with congenital cardiac diagnoses, as well as those requiring extracorporeal life support or left ventricular assist devices, were excluded from the analysis.

Patient and operative data elements were defined according to STS Adult Cardiac Database dictionary.^10^, ^11^, ^12^ Continuous variables are reported as median with interquartile range; categorical variables, as group proportion. Concomitant LAAC was identified using provided STS procedure and MAZE codes. Ligation method was stratified as epicardial suture, occlusion device, stapler, endocardial, or intra-atrial oversewing.

The primary outcome of interest was 30-day mortality. Major morbidity, defined as occurrence of pulmonary, infectious, hemorrhagic, or neurologic complications, were examined as a composite endpoint. Other outcomes of interest included 30-day all-cause readmission and risk-adjusted hospitalization costs. Cost data were captured by the International Classification of Diseases, Tenth Revision–based revenue codes and administrative charges. Cost categories were summative of indirect and direct costs for each phase of care. Cost records were then matched to the UCCSC data.

Patient and operative characteristics were compared between the LAAC group and non-LAAC group with the Wilcoxon rank-sum test for continuous variables and the χ^2^ test for categorical variables. The significance of temporal trends was determined using the Cochrane-Armitage test.^13^ Multilevel hierarchical logistic modeling was applied to delineate factors associated with LAAC while accounting for patient and operative characteristics. Bayesian estimates were applied to evaluate the risk- and reliability-adjusted probability of LAAC. The proportions of variance attributable to hospital and surgeon were calculated using the intraclass correlation coefficient. Separate two-level logistic models were developed to evaluate the associations of LAAC with inpatient mortality, major morbidity, nonhome discharge, 30-day readmission, and resource utilization. Generalized linear models were developed to perform risk-adjusted incremental cost analysis. Patient age stratified in accordance with CHADS2-VASc score, patient sex, operative urgency, history of prior cardiac operation, operative group, institution, year, and surgeon were used in risk-adjusted analyses.^14^ Subgroup analysis of outcomes of interest was performed based on a preoperative diagnosis of AF. Statistical analysis was performed using Stata 16.0 (StataCorp).

## Results

**Figure 1:**
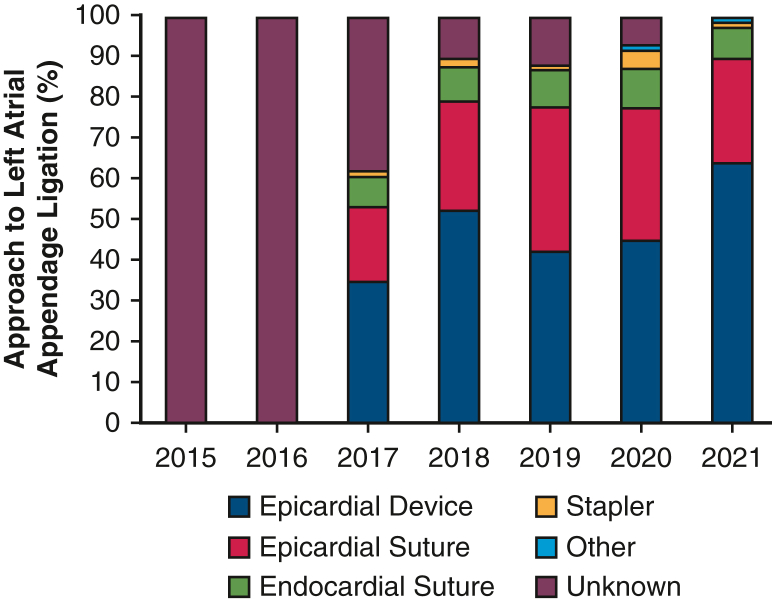
Modality of left atrial appendage closure data are not available for 2015 and 2016.

Among the 8699 patients who met the study criteria, 1377 (15.8%) underwent concomitant LAAC, of which 37.6% were performed for patients without a preoperative diagnosis of AF. The overall prevalence of preoperative AF in the study cohort rose steadily over time, from 16.7% in 2015 to 30.8% in 2021. The overall rate of LAAC was 15.8%, with the predominant methods entailing epicardial suture or device (Figure 1). The utilization of LAA closure increased from 7.0% in 2015 to 24.7% in 2021 (*P* < .001), with a similar increase in patients with preoperative AF (from 31.5% to 45.0%; *P* < .001).

Compared to the non-LAAC patients, the LAAC patients were older and more commonly female, with lower rates of smoking, insulin-dependent diabetes, preoperative cerebrovascular disease, and peripheral arterial disease (Table 1). The median CHADS-VASC2 score was similar for LAAC and non-LAAC patients (2; IQR, 4), while the STS predicted risk of mortality (PROM) was modestly higher for the LAAC cohort (1.3% vs 1.0%; *P* < .001). Stratification by preoperative AF status revealed a similar association with age at LAAC, sex, and smoking (Table E1). The most commonly performed concomitant procedure was mitral valve repair, followed by mitral valve replacement (Table E2). The overall rate of surgical ablation for patients with preoperative AF was 24.1%, with 67.8% of LAAC patients undergoing concomitant ablation (Table E1). Considering all patients undergoing surgical ablation with preoperative AF, 88.6% underwent LAAC. Surgical ablation was reported in 1.6% of patients without a preoperative diagnosis of AF.

**Table 1:** Overall demographics and clinical characteristics

Characteristic	Non-LAAC (N = 7322)	LAAC (N = 1377)	*P* value
Age, y, median (IQR)	63 (55-71)	67 (59-74)	<.001
Female sex, n (%)	2034 (27.8)	428 (31.1)	.013
Body mass index, kg/m^2^, median (IQR)	27.2 (24.0-30.9)	26.4 (23.4-30.1)	<.001
Caucasian race, n (%)	3290 (59.8)	588 (66.3)	<.001
Preoperative atrial fibrillation, n (%)	1871 (25.5)	859 (62.4)	<.001
Tobacco use, n (%)	905 (12.4)	105 (7.6)	<.001
Prior myocardial infarction, n (%)	2202 (30.3)	303 (22.1)	<.001
Insulin-dependent diabetes, n (%)	896 (12.2)	122 (8.9)	<.001
Peripheral vascular disease, n (%)	724 (10.0)	112 (8.2)	.034
Dialysis-dependent renal failure	523 (7.1)	76 (5.5)	.029
Cerebrovascular disease, n (%)	1256 (17.3)	222 (16.2)	.326
Prior stroke, n (%)	694 (9.5)	153 (11.1)	.061
Moderate to severe chronic lung disease, n (%)	521 (7.1)	96 (6.9)	.849
Preoperative anemia (Hct ≤30), n (%)	999 (13.6)	143 (10.4)	.001
Malnutrition (albumin <3.5), n (%)	1472 (20.1)	249 (18.1)	.084
Aortic insufficiency (>mild), n (%)	1326 (18.1)	217 (15.8)	.036
Aortic stenosis (>mild), n (%)	1476 (20.2)	253 (18.4)	.13
Mitral regurgitation (>mild), n (%)	1295 (17.7)	737 (53.5)	<.001
Mitral stenosis (>mild), n (%)	265 (3.6)	138 (10.0)	<.001
Tricuspid regurgitation (>mild), n (%)	773 (10.6)	326 (23.7)	<.001
Intra-aortic balloon pump, n (%)	376 (5.1)	67 (4.9)	.49
Reoperative status, n (%)	927 (12.7)	125 (9.1)	<.001
Prior valve surgery, n (%)	659 (9.0)	106 (7.7)	.18
Mitral repair, n (%)	542 (7.4)	426 (30.9)	<.001
Surgical ablation, n (%)	95 (1.3)	655 (47.6)	<.001
Preoperative ejection fraction, %, median (IQR)	60 (50-65)	59 (50-63)	.73
Predicted morbidity/mortality, %, median (IQR)	1.02 (0.5-2.2)	1.26 (0.5-2.9)	<.001
Status, n (%)			<.001
Elective	3807 (52.0)	891 (64.7)	
Emergent	450 (6.2)	19 (1.4)	
Emergent salvage	25 (0.3)	4 (0.3)	
Urgent	3040 (41.5)	463 (33.6)	

*LAAC*, Left atrial appendage closure; *IQR*, interquartile range.

**Figure 2:**
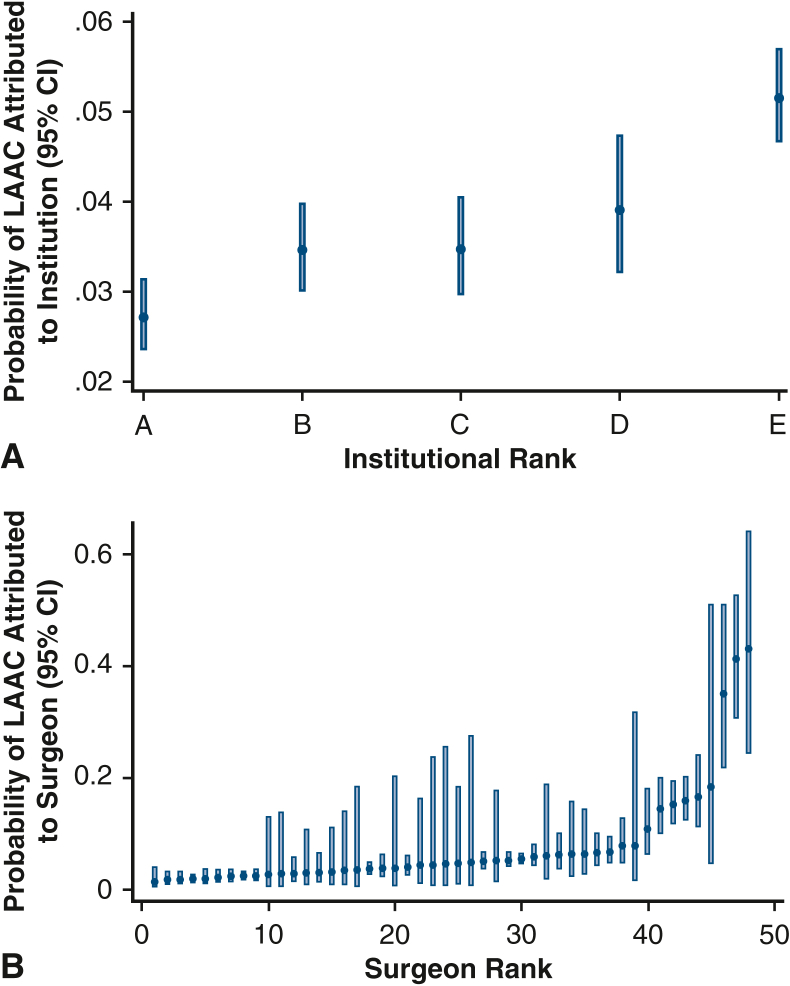
Risk-adjusted probability of left atrial appendage closure attributed to institution (A) and surgeon (B), demonstrating a greater magnitude of variation attributable to surgeon compared to institution. Each estimate reported with its 95% confidence interval.

Although the institutional rate of LAAC ranged from 12.9% to 21.1%, analysis of intraclass correlation revealed that 1.3% (95% CI, 0.3%-5.1%) of the variation was attributable to institution-level factors (Figure 2, *A*). Surgeon rates of LAAC ranged from 2.3% to 66.7%, with 26.4% of the variation in rate explained by surgeon variation (Figure 2, *B*).

**Figure 3:**
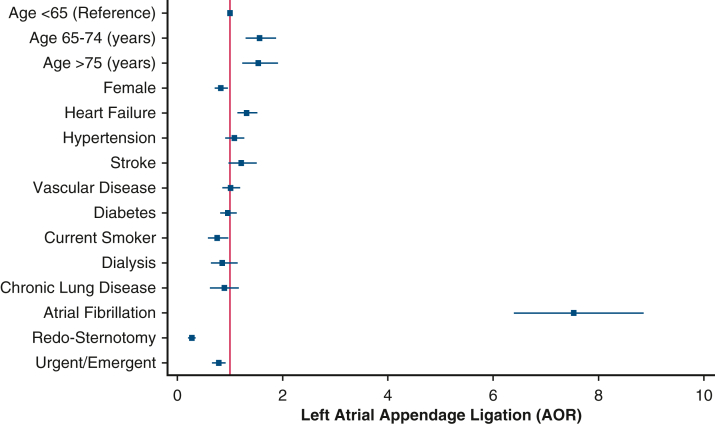
Risk-adjusted patient and operative characteristics associated with left atrial appendage closure. The *red line* represents the reference group. Age stratification was done according to CHADs-VASC2 cutoffs. STS factors include heart failure (clinical signs or symptoms of heart failure within 2 weeks of surgery). Composite variables were generated according to the following: stroke (any prior stroke with symptoms that did not resolve within 24 hours, recent or remote), vascular disease (history of peripheral vascular disease, prior myocardial infarction, aortic calcification), diabetes (preoperative A1C >6.5%, fasting glucose ≥126 mg/dL, random plasma glucose ≥200 mg/dL), dialysis (undergoing dialysis prior to surgery), chronic lung disease (moderate to severe), and urgent/emergent (urgent, emergent, emergent salvage) patient clinical status.

Several factors, including older age, heart failure, and preoperative history of AF, were associated with increased odds of LAA (Figure 3). Female sex, history of prior sternotomy, and urgent/emergent operative status were linked to lower risk-adjusted odds of concomitant LAAC (Figure 3). With isolated CABG as the reference, aortic valve and aortic procedures were less frequently associated with LAAC, whereas all other categorized mitral and tricuspid procedures were associated with greater odds of LAAC (Table E3).

On adjusted analysis, LAAC patients experienced longer cardiopulmonary bypass and aortic cross-clamp times compared to non-LAAC patients (Table 2). Stratification by operative subtype showed no significant difference in cardiopulmonary bypass or aortic cross-clamp times in the LAAC cohort except for isolated CABG, isolated mitral valve replacement, and mitral valve repair (Table E2). Although the difference for isolated CABG reached statistical significance, there was only a 5-minute difference in median bypass times and a 4-minute difference in median cross-clamp times (Table 2). Longer cross-clamp and cardiopulmonary bypass times were observed among patients undergoing isolated mitral valve replacement with concomitant ablation. The operative time among patients undergoing mitral valve replacement or mitral valve repair without ablation was not evaluated, owing to a lack of sufficient sample size in the LAAC group.

**Table 2:** Unadjusted outcomes stratified by LAAC

Unadjusted outcome	Non-LAAC (N = 7322)	LAAC (N = 1377)	*P* value
30-d mortality, n (%)	264 (3.7)	56 (4.1)	.46
STS major morbidity, n (%)∗	1331 (18.2)	216 (15.7)	.03
Postoperative stroke, n (%)	207 (2.8)	29 (2.1)	.13
Postoperative atrial fibrillation, n (%)	1911 (26.1)	435 (31.6)	<.001
Prolonged ventilation, n (%)	900 (12.3)	151 (10.9)	.17
Postoperative dialysis, n (%)	266 (3.6)	521 (3.7)	.30
Deep sternal wound infection, n (%)	28 (0.4)	1 (0.07)	.07
Red blood cell transfusion (perioperatively), n (%)	3733 (51.0)	654 (47.5)	.02
Any blood product postoperatively, n (%)	2860 (39.3)	512 (37.2)	.15
Reoperation, bleeding, n (%)	272 (3.7)	41 (3.0)	.18
Postoperative intubation time, h, median (IQR)	5.5 (3.5-11.8)	5.2 (3.5-10.4)	.069
ICU duration, h, median (IQR)	77.2 (48-123)	93 (59-145)	<.001
Length of stay, d, median (IQR)	9 (6-16)	10 (6-15)	.27
Costs, 1K$, median (IQR)			
Operating room	26 (21-34)	33 (28-42)	<.001
Intensive care unit	18 (11-32)	22 (14-37)	<.001
Imaging	2.3 (1.3-4.1)	2.2 (1.4-3.9)	.12
Laboratory	3.6 (2.4-5.6)	4.1 (2.9-6.1)	<.001
Pharmacy	5.0 (3.1-8.6)	4.9 (3.3-8.3)	.72
Respiratory therapy	1.9 (1.1-3.9)	1.9 (1.1-3.9)	.35
Nonhome discharge, n/N (%)	1214/7003 (17.4)	215/1309 (16.4)	.42
30-d readmission, n/N (%)	741/6737 (11.0)	133/1266 (10.6)	.61

*LAAC*, Left atrial appendage closure; *STS*, Society of Thoracic Surgeons; *IQR*, interquartile range; *ICU*, intensive care unit.

∗ STS major morbidity is defined as prolonged postoperative ventilation, deep sternal wound infection, postoperative stroke, postoperative arrest, renal failure necessitating dialysis, or reoperation for any reason.

Comparison of the LAAC and non-LAAC groups revealed comparable unadjusted rates of inpatient mortality, postoperative stroke, prolonged ventilation, blood product use, and reoperation for bleeding (Table 2). Rates of new-onset postoperative AF were higher in the LAAC group (31.6% vs 26.1%; *P* < .001). The duration of postoperative intubation and hospital length of stay were comparable, despite a longer ICU length of stay for the LAAC group (Table 2). Discharge to rehabilitation or a skilled nursing facility (ie, nonhome discharge), as well as readmission within 30 days, were similar in the 2 groups (Table 2). Risk-adjusted analysis revealed no significant associations between LAAC and inpatient mortality, new-onset postoperative AF, and 30-day readmission (Table 3). Further stratification by method of LAAC and by device versus suture (intracardial or epicardial ligation) in risk-adjusted models of new-onset postoperative AF demonstrated no association with LAAC method and onset of this postoperative complication (Table E4).

**Table 3:** LAAC parameter estimates from multivariate hierarchical regression analysis of postoperative outcomes

Outcomes and resource use	OR/point estimate	95% CI
Outcome		
30-d mortality	1.02	0.72-1.44
STS major morbidity∗	0.77	0.99-1.00
Postoperative atrial fibrillation†	1.00	0.87-1.16
30-d readmission	1.49	0.91-2.46
Resource use		
Cardiopulmonary bypass time, min	8.36	0.22-16.5
Aortic cross-clamp time, min	5.07	−0.55 to 10.7
Overall length of stay, d	0.23	−0.48 to 0.93
ICU length of stay, h	9.55	−8.84 to 27.9

*OR*, Odds ratio; *CI*, confidence interval; *STS*, Society of Thoracic Surgeons; *ICU*, intensive care unit; *LAAC*, left atrial appendage closure.

∗ STS major morbidity is defined as prolonged postoperative ventilation, deep sternal wound infection, postoperative stroke, postoperative arrest, renal failure necessitating dialysis, or reoperation for any reason.

† Identified by the STS variable COTAFIB, which is defined by atrial fibrillation/flutter requiring treatment, excluding patients who were in atrial fibrillation at the start of the surgery.

**Figure E1:**
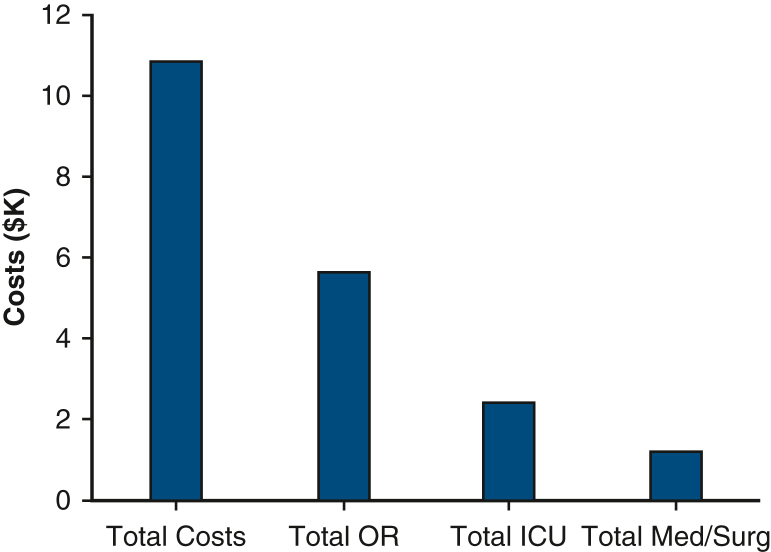
Left atrial appendage closure cost contribution stratified by phase of care. *LAAC*, Left atrial appendage closure; *ICU*, intensive care unit.

After risk adjustment for patient and operative facets (age, STS PROM, history of redo sternotomy, patient clinical status, operative intervention, institution, surgeon, and occurrence of major postoperative morbidity), LAAC was associated with a significant increase in hospitalization costs ($+10,602; 95% CI, $4078-$17,126). Breakdown of the cost components revealed greatest contribution from the operative phase of care (Figure E1). Given the potential collinearity between these factors and STS PROM score, we performed a cost model sensitivity analysis including only PROM, institution, surgeon, and occurrence of postoperative complications and found a smaller, yet significant incremental increase in costs ($+6581; 95% CI, $314-12,848). After stratification by LAAC method, no statistically significant cost difference was noted, with epicardial suture ligation as the reference (Table E5). Sensitivity analyses of examined outcomes stratified by preoperative AF diagnosis were performed, and the results are presented in Tables E6 and E7. Regardless of preoperative AF diagnosis, 30-day mortality was comparable, but rates of major morbidity were lower for patients who underwent concomitant LAAC (Table E6). Unadjusted rate- and risk-adjusted odds of postoperative AF were higher among patients without a preoperative diagnosis of AF (Tables E6 and E7). Unadjusted total costs were higher in the LAAC group irrespective of preoperative AF diagnosis, with the largest-magnitude difference in the operative cost category (Table E6). After risk adjustment, the duration of cardiopulmonary bypass time was longer for LAAC patients stratified by preoperative AF status (Table E7). Notably, LAAC was associated with lower odds of 30-day mortality and major STS morbidity among patients with a preoperative diagnosis of AF (Table E7).

## Discussion

**Figure 4:**
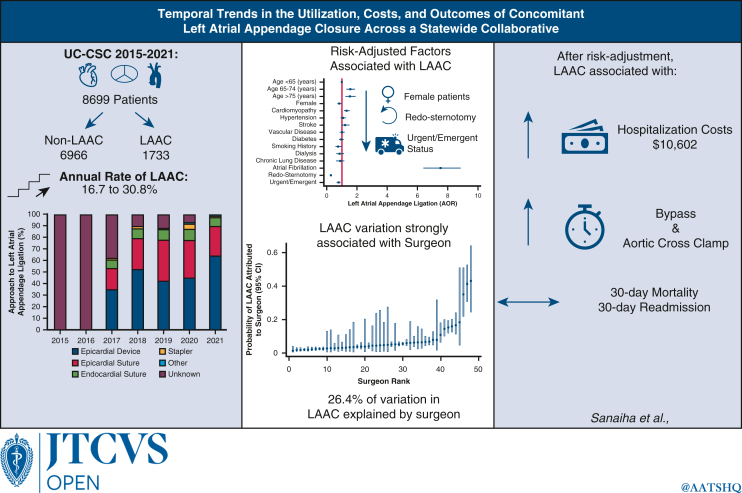
Graphical abstract. *UC-CSC*, University of California Cardiac Surgery Consortium; *LAAC*, left atrial appendage closure.

As the clinical impact of new-onset postoperative AF is further elucidated, management of the LAA at the time of cardiac surgery is an area of growing interest and controversy.^7^ The present analysis of our statewide academic cardiac surgery consortium underscores several important aspects of LAAC and the associations with patient demographics, clinical characteristics, operative details, and postoperative outcomes (Figure 4). Among the 8699 patients undergoing coronary or valve operations, the rate of LAAC has increased steadily. Female sex is associated with lower risk-adjusted odds of LAAC. Not unexpectedly, variation in LAAC was driven to a greater extent by surgeon than by institution. LAAC was associated with increased bypass and aortic cross-clamp times with higher hospitalization costs, except when accounting for the method of LAAC. Further exploration of the key findings and their implications is warranted.

Our analysis reveals a significant rise in the prevalence of preoperative AF over the study period, consistent with other global reports of its increased prevalence and indicating a growing need for interventions targeting AF-related complications such as stroke.^15^ The observed increase in the annual rate of LAAC reflects a corresponding escalation in the adoption of this procedure, particularly for patients with preexisting AF.^7^^,^^16^ Our findings parallel other statewide analyses examining the increased use of LAAC for AF patients during cardiac surgery.^17^

With the increasing prevalence of AF and use of LAAC, we were interested in the impact of surgeon and institution on concomitant LAAC. We found greater variation attributed to surgeon, with a smaller contribution from institution. Although our present analysis demonstrates significant surgeon-related variation, others have reported that institutional variation also exists, suggesting variability of LAAC utilization at multiple levels.^17^

Data on short- and long-term closure, which is largely dependent on surgical technique, is limited in the literature to selected institutional studies and more recent clinical trials. One of the earliest studies examining LAAC reported a high rate of failure (60%).^18^ This single-institution study reported data from early generations of device closure and ultimately advocated for surgical excision. Such findings are difficult to apply as the clinical practice has shifted away from excision to intracardiac or epicardial closure with either suture or a device. A more recent institutional study of appendage clipping reported a 92% rate of successful closure.^19^ While the STS Adult Cardiac Surgery Database includes limited echocardiographic fields, future iterations perhaps may collect specific echocardiographic metrics, such as residual stump or flow, at the end of applicable procedures. In summary, additional prospective studies with robust echocardiographic and long-term clinical data are warranted to better delineate the association with outcomes with success of left atrial appendage closure.

The demographic and clinical profile of patients undergoing concomitant LAAC differed from those not undergoing LAAC. Although the LAAC recipients were more frequently female, the risk-adjusted odds of LAAC was lower in women. Beyond the present statewide analysis, lower rates of concomitant LAAC in female patients undergoing valve surgery also has been shown at a national level.^20^ Given that AF has been associated with higher all-cause mortality and greater risk of stroke in women compared to men, the disparities in concomitant LAAC in females needs to be closely examined.^21^

Risk-adjusted analysis also identified older age, heart failure, and preoperative AF as factors associated with concomitant LAAC, whereas prior sternotomy, and urgent/emergent surgery were inversely correlated. Specific cardiac procedures, particularly mitral and tricuspid interventions, exhibited a strong association with LAAC, consistent with other institutional reports of routine LAAC at time of mitral repair in patients without AF.^22^ Although it is understandable that prior sternotomy was inversely associated with LAAC because of the technical complexity of pericardial adhesiolysis, the decreased use among patients with insulin-dependent diabetes and those with renal failure highlight opportunities for highest-yield intervention.^23^, ^24^, ^25^, ^26^ Often these patients with significant chronic cardiovascular comorbidities are those at greatest risk for thromboembolic complications of AF, and thus a more concerted effort at considering LAAC is warranted. Furthermore, as societal guidelines expand indications for LAA management and surgical ablation, surveillance for guideline-concordant care will be paramount.

While the literature has extensively examined the association of LAAC with such perioperative outcomes as stroke, inpatient mortality, and rebleeding, the present analysis is one of the first to evaluate the impact of LAAC on bypass and cross-clamp duration by operative subtype.^16^^,^^17^ Differences in operative time between the LAAC and non-LAAC groups were not consistently significant across all surgical subtypes, suggesting nuanced considerations in patient selection and procedural planning. For isolated CABG, the attributable contribution of LAAC to operative time was up to 5 minutes. Among patients undergoing mitral valve replacement without surgical ablation, we found increased bypass and aortic cross-clamp times regardless of LAAC method. The association between LAAC and increased cardiopulmonary bypass and aortic cross-clamp times underscores the potential impact on operative logistics. Quantifying the impact of LAAC on operative duration will aid evaluation of the clinical value of LAAC among patients with AF and those without AF.

Importantly, despite differences in operative characteristics, LAAC was not associated with higher rates of short-term adverse outcomes such as mortality, stroke, or prolonged ventilation, consistent with several other reports.^16^^,^^17^ Furthermore, in the present analysis, LAAC recipients had a higher incidence of new-onset postoperative AF compared to non-LAAC patients; however, this difference did not achieve statistical significance in a risk-adjusted model including all patients regardless of preoperative AF status. A sensitivity analysis of only patients without preoperative AF demonstrated a risk-adjusted increase in the odds of AF. The unadjusted rate and sensitivity analysis of a limited cohort without preoperative AF is consistent with other reports in the literature suggesting that LAAC may be associated with an increased incidence of postoperative AF.^16^ Despite a longer ICU length of stay for LAAC recipients, the overall hospital length of stay and postdischarge outcomes were similar in the 2 groups. Therefore, LAAC did not seem to significantly impact hospital throughput.

Beyond differences in operative time, LAAC was found to incur an incremental increase in total hospitalization costs, driven primarily by patient characteristics, institutional factors, and postoperative complications. The cost difference in the present analysis is greater than reports in the literature^16^; however, this cost differential was attenuated when adjusting for method of LAAC. In the United States alone, new-onset postoperative AF affects 100,000 patients annually, with an associated hospital cost of $10,000 to $11,500 per patient. These estimates, taken together with American Heart Association data estimating a 30% incidence of postoperative AF, suggest expenditures to exceed $2 billion per year.^2^^,^^27^ Thus, while randomized clinical trial data on prophylactic LAAC are pending, the incremental increase in hospitalization costs of approximately $10,000 in patients without AF underscores the importance of identifying the patient phenotype that derives the greatest benefit from this intervention.

Although our analysis provides a pragmatic review of trends in use across a large academic health system, there are several limitations to consider. The STS adult cardiac surgery database provides a comprehensive range of patient and operative characteristics used for risk adjustment; however, we acknowledge the retrospective nature of the study, which might not fully capture treatment selection bias and deviations from societal guidelines. Furthermore, data extraction is dependent on variability in documentation of comorbidities and operative technique. Although the method of LAAC is documented, variation remains among surgeons in technique and devices used, which may impact operative times and long-term outcomes. Additional limitations are related to the data extraction methods of the consortium. We were unable to examine outcomes beyond 30 days or echocardiographic data confirming successful LAAC. Finally, we acknowledge that our analysis is based on data extracted from a multi-institutional consortium within a single state, focusing on academic cardiac surgical practices, which may limit the generalizability of our findings.

In conclusion, our findings elucidate the evolving landscape of LAAC use, patient selection criteria, procedural intricacies, and economic implications. Although LAAC is associated with some increased procedural complexity and costs, emerging evidence of potential benefits in prevention of thromboembolic complications with minimal associated morbidity support ongoing consideration in routine cardiac operations. Further research is warranted to refine patient selection criteria, address disparities in application, optimize procedural techniques, and evaluate long-term clinical outcomes and cost-effectiveness.

## Conflict of Interest Statement

Dr Madani reported consulting for Bayer, Wexler Surgical, and Actelion Pharmaceuticals. Dr Shemin reports consulting for Edwards Lifesciences. Dr Benharash reported consulting for Atricure. All other authors reported no conflicts of interest.

The *Journal* policy requires editors and reviewers to disclose conflicts of interest and to decline handling or reviewing manuscripts for which they may have a conflict of interest. The editors and reviewers of this article have no conflicts of interest.

## Figures and Tables

**Table E1:** Demographic and clinical characteristics stratified by preoperative atrial fibrillation status

Characteristic	Without preoperative atrial fibrillation			Preoperative atrial fibrillation		
	Non-LAAC (N = 5451)	LAAC (N = 518)	*P* value	Non-LAAC (N = 1871)	LAAC (N = 859)	*P* value
Age, y, median (IQR)	63 (54-70)	65 (56-72)	.004	64 (55-72)	68 (61-75)	<.001
Female sex, n (%)	1469 (27.0)	163 (31.5)	.027	565 (30.2)	265 (30.9)	.73
BMI, kg/m^2^, median (IQR)	27.2 (24.0-30.8)	25.7 (23.0-29.0)	<.001	27.2 (24.0-31.2)	26.9 (23.7-30.5)	.34
Caucasian race, n (%)	2329 (57.3)	198 (65.8)	.004	961 (66.9)	390 (66.6)	.86
Tobacco use, n (%)	702 (12.9)	43 (8.3)	.003	203 (10.9)	62 (7.2)	.003
Prior myocardial infarction, n (%)	1861 (34.4)	117 (22.6)	<.001	341 (18.5)	186 (21.7)	.05
Insulin-dependent diabetes, n (%)	756 (13.9)	47 (9.1)	.002	140 (7.5)	75 (8.8)	.26
Peripheral vascular disease, n (%)	543 (10.1)	44 (8.9)	.27	181 (9.9)	68 (7.9)	.11
Dialysis dependence, n (%)	417 (7.7)	37 (7.1)	.68	106 (5.7)	39 (4.5)	.22
Cerebrovascular disease, n (%)	917 (16.9)	64 (12.4)	.01	339 (18.4)	158 (18.5)	.95
Prior stroke, n (%)	486 (8.9)	41 (7.9)	.44	208 (11.1)	112 (13.0)	.15
Moderate-severe lung disease, n (%)	384 (7.0)	29 (5.6)	.22	137 (7.3)	67 (7.8)	.66
Preoperative anemia (Hct ≤30), n (%)	723 (13.3)	53 (10.2)	.05	276 (14.8)	90 (10.5)	.002
Malnutrition (albumin <3.5), n (%)	1092 (20.0)	84 (16.2)	.04	380 (20.3)	165 (19.2)	.50
Aortic insufficiency (>mild), n (%)	586 (10.8)	30 (5.8)	<.001	740 (40.0)	187 (21.8)	<.001
Aortic stenosis (>mild), n (%)	764 (14.0)	45 (8.6)	.001	712 (38.1)	208 (24.2)	<.001
Mitral regurgitation (>mild), n (%)	917 (16.8)	308 (59.5)	<.001	378 (20.2)	429 (49.9)	<.001
Mitral stenosis (>mild), n (%)	154 (2.8)	33 (6.4)	<.001	111 (5.9)	105 (12.2)	<.001
Tricuspid regurgitation (>mild), n (%)	481 (8.8)	82 (15.8)	<.001	292 (15.6)	244 (28.4)	<.001
Intra-aortic balloon pump, n (%)	309 (5.7)	35 (6.8)	.31	67 (3.6)	32 (3.7)	.86
Reoperative status, n (%)	463 (8.5)	31 (5.9)	.05	464 (24.8)	94 (10.9)	<.001
Prior valve surgery, n (%)	282 (5.2)	25 (4.8)	.73	377 (20.2)	81 (9.4)	<.001
Mitral repair, n (%)	438 (8.0)	212 (40.9)	<.001	104 (5.6)	214 (24.9)	<.001
Surgical ablation, n (%)	20 (0.4)	73 (14.1)	<.001	75 (4.0)	682 (67.8)	<.001
Predicted mortality, %, median (IQR)	0.1 (0.5-2.0)	0.9 (0.5-2.3)	.36	1.5 (0.7-3.5)	1.6 (0.7-3.3)	.33
Status, n (%)			<.001			<.001
Elective	2710 (49.7)	337 (65.1)		1097 (58.6)	554 (64.5)	
Emergent	269 (4.9)	10 (1.9)		181 (9.7)	9 (1.1)	
Emergent salvage	16 (0.3)	2 (0.4)		9 (0.5)	2 (0.2)	
Urgent	2456 (45.1)	169 (32.6)		584 (31.2)	294 (34.2)	

*LAAC*, Left atrial appendage closure; *BMI*, body mass index; *IQR*, interquartile range.

**Table E2:** Median cardiopulmonary bypass and aortic cross-clamp times stratified by operative type

Operative group	Proportion of LAAC (N = 1377),%	Cardiopulmonary bypass time			Aortic cross clamp time		
		Non-LAAC	LAAC	*P* value	Non-LAAC	LAAC	*P* value
AVR + CABG	4.0	167 (132-203)	166 (133-199)	.948	131 (101-154)	128 (100-155)	.403
Isolated AVR	5.1	117 (96-146)	122 (93-144)	.614	83 (68-102)	85 (66-97)	.77
Isolated AVR + isolated MVR	4.7	212 (178-258)	201 (169-241)	.282	159 (132-187)	155 (127-183)	.67
Isolated CABG	20.9	118 (96-142)	123 (99-153)	.02	89 (70-108)	93 (69-113)	.04
Isolated MVR	11.2	137 (112-176)	151 (125-197)	.01	90 (73-111)	100 (84-120)	.004
MV repair	22.7	151 (123-180)	177 (140-221)	<.001	94 (79-118)	110 (93-133)	<.001
MV repair + CABG	4.4	181 (157-212)	193 (162-228)	.615	140 (121-156)	148 (123-165)	.297
MVR + CABG	3.9	198 (167-246)	199 (161-230)	.852	142 (116-163)	137 (107-167)	.576
Aortic procedure	12.7	201 (155-262)	209 (164-266)	.36	132 (100-171)	143 (115-181)	.01
Tricuspid valve∗	10.5	181 (135-247)	196 (149-246)	.116	111 (63-151)	123 (88-156)	.149

*LAAC*, Left atrial appendage closure; *AVR*, aortic valve replacement; *CABG*, coronary artery bypass grafting; *MVR*, mitral valve replacement; *MV*, mitral valve.

∗ Tricuspid valve procedures include repair or replacement with or without additional valve or coronary bypass procedures.

**Table E3:** Rate of LAAC and risk-adjusted odds of LAAC by operative subtype

Operative subtype	Non-LAAC	LAAC	OR	95% CI
Isolated CABG	3237 (91.8)	288 (8.2)	Reference	Reference
AVR-CABG	343 (86.2)	55 (13.8)	0.59	0.42-0.84
Isolated AVR	759 (91.6)	70 (8.4)	0.44	0.32-0.61
AVR-MVR	89 (57.8)	65 (42.2)	3.86	2.52-5.92
Isolated MVR	258 (62.6)	154 (37.4)	7.27	5.31-9.94
MV repair	381 (55.0)	312 (45.0)	8.06	6.24-10.42
MV repair + CABG	76 (55.9)	60 (44.1)	8.18	5.38-12.4
MVR + CABG	63 (54.3)	53 (45.7)	6.65	0.31-0.51
Aortic procedure	1606 (90.1)	175 (9.8)	0.40	1.89-3.38
Tricuspid valve∗	510 (77.9)	145 (22.1)	2.52	1.88-3.38

*LAAC*, Left atrial appendage closure; *OR*, odds ratio; *CI*, confidence interval; *CABG*, coronary artery bypass grafting; *AVR*, aortic valve replacement; *MVR*, mitral valve replacement; *MV*, mitral valve.

∗ Tricuspid valve procedures include repair or replacement with or without additional valve or coronary bypass procedures.

**Table E4:** Risk-adjusted∗ odds of new onset postoperative atrial fibrillation by LAAC method using generalized linear modeling

LAAC method	Adjusted OR	95% CI
Epicardial suture ligation	Reference	
Epicardial occlusion device	0.90	0.60-1.36
Epicardial stapler	0.14	0.02-1.12
Endocardial suture	0.79	0.41-1.53
Suture ligation (intra-atrial vs epicardial)†	Reference	
Epicardial device	0.84	0.63-1.10

*LAAC*, Left atrial appendage closure; *OR*, odds ratio; *CI*, confidence interval.

∗ Risk adjustment performed with the CHADS-VASC2 score.

† Separate risk-adjusted model group all methods of suture ligation to epicardial device.

**Table E5:** Risk-adjusted relative cost contribution by LAAC method using generalized linear modeling

LAAC method	Point estimate, $	95% CI
Epicardial suture ligation	Reference	
Epicardial occlusion device	−11,935	−27,456 to 3586
Stapler	−15,405	−59,047 to 28,237
Endocardial suture	−8223	−32,189 to 15,743
Other	4146	−93,078 to 101,369

*LAAC*, Left atrial appendage closure; *CI*, confidence interval.

**Table E6:** Unadjusted outcomes stratified by LAAC stratified by preoperative atrial fibrillation status

Unadjusted outcome	Without atrial fibrillation			With atrial fibrillation		
	Non-LAAC (N = 5451)	LAAC (N = 518)	*P* value	Non-LAAC (N = 1871)	LAAC (N = 859)	*P* value
30-d mortality, n (%)	151 (2.8)	15 (2.9)	.95	113 (6.1)	41 (4.8)	.19
STS major morbidity, n (%)∗	869 (15.9)	62 (11.9)	.02	462 (24.7)	154 (17.9)	<.001
Postoperative stroke, n (%)	141 (2.6)	9 (1.7)	.24	66 (3.5)	20 (2.3)	.096
Postoperative atrial fibrillation, n (%)	1334 (24.5)	201 (38.8)	<.001	–	–	--
Prolonged ventilation, n (%)	550 (10.1)	37 (7.1)	.03	350 (18.7)	114 (13.3)	<.001
Postoperative dialysis, n (%)	162 (3.0)	14 (2.7)	.73	104 (5.6)	37 (4.3)	.17
Sternal wound infection, n (%)	24 (0.4)	0	.13	4 (0.21)	1 (0.12)	.58
RBC transfusion (perioperatively), n (%)	2651 (48.7)	241 (46.5)	.36	1082 (57.8)	413 (48.1)	<.001
Any blood product postoperatively, n (%)	1987 (36.7)	172 (33.3)	.12	873 (46.9)	340 (39.6)	<.001
Reoperation, bleeding, n (%)	172 (3.2)	10 (1.9)	.12	100 (5.3)	31 (3.6)	.05
Postoperative intubation, h, median (IQR)	5 (3-10)	4 (3-7)	<.001	6.2 (3.9-16.2)	5.8 (3.8-12.1)	.04
ICU length of stay, h, median (IQR)	74 (47-119)	85 (53-123)	.12	93 (54-149)	99.6 (63.5-161)	.03
Overall length of stay, d, median (IQR)	9 (6-15)	10 (5.5-16)	.12	9.5 (6-16)	9 (6-15)	.47
Costs, 1K$, median (IQR)	78.3 (59.7-111.2)	82.4 (66.7-109.8)	.006	82.9 (62.5-121)	95.3 (72.3-130.1)	<.001
Operating room	25.0 (20.1-30.7)	30.5 (25.5-36.7)	<.001	26.8 (21.4-34.5)	33.3 (27.9-41.6)	<.001
ICU	17.4 (9.7-30.2)	19.3 (13.0-32.0)	.001	18.5 (10.6-32.2)	22.8 (14.1-37.1)	<.001
Imaging	2.2 (1.3-4.0)	1.9 (1.3-3.4)	.001	2.3 (1.4-4.1)	2.2 (1.4-4.0)	.11
Laboratory	3.4 (2.3-5.2)	3.8 (2.9-5.4)	.001	3.6 (2.4-5.6)	4.1 (2.9-6.1)	<.001
Pharmacy	4.8 (2.9-7.9)	4.4 (3.0-7.2)	.02	5.0 (3.1-8.6)	4.9 (3.3-8.3)	.70
Respiratory therapy	1.9 (1.1-3.6)	1.6 (1.0-2.9)	.002	2.0 (1.1-3.9)	1.9 (1.1-3.9)	.33
Nonhome discharge	854/5265 (16.2)	68/500 (13.6)	.13	360/1738 (20.7)	147/809 (18.2)	.14

*LAAC*, Left atrial appendage closure; *STS*, Society of Thoracic Surgeons; *RBC*, red blood cell; *IQR*, interquartile range; *ICU*, intensive care unit.

∗ STS major morbidity is defined as prolonged postoperative ventilation, deep sternal wound infection, postoperative stroke, postoperative arrest, renal failure necessitating dialysis, or reoperation for any reason.

**Table E7:** LAAC parameter estimates from multivariate hierarchical regression analysis of postoperative outcomes stratified by preoperative atrial fibrillation diagnosis

Outcomes and resource use	Without preoperative atrial fibrillation		Preoperative atrial fibrillation	
	OR/point estimate	95% CI	OR/point estimate	95% CI
Outcome				
30-d mortality	1.18	0.65-2.16	0.88	0.57-1.35
STS major morbidity∗	0.78	0.57-1.07	0.75	0.59-0.96
Postoperative atrial fibrillation†	1.52	1.23-1.89	–	–
30-d readmission	1.58	0.61-4.09	1.65	0.87-3.18
Resource use				
Cardiopulmonary bypass time, min	17	1-33	11	0.5-23
Aortic cross-clamp time, min	9.8	−1 to 21	9	1-16
Overall length of stay, d	0.75	−0.3 to 1.8	−0.1	−1 to 0.8
ICU length of stay, h	33.9	−4.23 to 71.9	5.3	−18.9 to 29.4

*OR*, Odds ratio; *CI*, confidence interval; *STS*, Society of Thoracic Surgeons; *ICU*, intensive care unit; *LAAC*, left atrial appendage closure.

∗ STS major morbidity defined as prolonged postoperative ventilation, deep sternal wound infection, postoperative stroke, postoperative arrest, renal failure requiring dialysis, or reoperation for any reason.

† Identified by the STS variable COTAFIB, which is defined by atrial fibrillation/flutter requiring treatment, excluding patients who were in atrial fibrillation at the start of the surgery.
